# Dietary fiber prevents obesity-related liver lipotoxicity by modulating sterol-regulatory element binding protein pathway in C57BL/6J mice fed a high-fat/cholesterol diet

**DOI:** 10.1038/srep15256

**Published:** 2015-10-29

**Authors:** Shufen Han, Jun Jiao, Wei Zhang, Jiaying Xu, Zhongxiao Wan, Weiguo Zhang, Xiaoran Gao, Liqiang Qin

**Affiliations:** 1 Department of Nutrition and Food Hygiene, Jiangsu Key Laboratory of Preventive and Translational Medicine for Geriatric Diseases, School of Public Health, Soochow University, 199 Renai Road, Suzhou City 215123, China; 2 Key Laboratory of Radiation Biology, School of Radiation Medicine and Protection, Soochow University, 199 Renai Road, Suzhou City 215123, China; 3 DSM Nutritional Products Human Nutrition and Health, Unite 5-7, Floor 8, Tower C, Parkview Green, 9 Dongdaqiao Road, Beijing 100020, China; 4 DSM Nutritional Products Human Nutrition and Health, 476 Libing Road, Shanghai 201203, China

## Abstract

Adequate intake of dietary fibers has proven metabolic and cardiovascular benefits, molecular mechanisms remain still limited. This study was aimed to investigate the effects of cereal dietary fiber on obesity-related liver lipotoxicity in C57BL/6J mice fed a high-fat/cholesterol (HFC) diet and underlying mechanism. Forty-eight adult male C57BL/6J mice were randomly given a reference chow diet, or a high fat/choleserol (HFC) diet supplemented with or without oat fiber or wheat bran fiber for 24 weeks. Our results showed mice fed oat or wheat bran fiber exhibtied lower weight gain, lipid profiles and insulin resistance, compared with HFC diet. The two cereal dietary fibers potently decreased protein expressions of sterol regulatory element binding protein-1 and key factors involved in lipogenesis, including fatty acid synthase and acetyl-CoA carboxylase in target tissues. At molecular level, the two cereal dietary fibers augmented protein expressions of peroxisome proliferator-activated receptor alpha and gamma, liver X receptor alpha, and ATP-binding cassette transporter A1 in target tissues. Our findings indicated that cereal dietary fiber supplementation abrogated obesity-related liver lipotoxicity and dyslipidemia in C57BL/6J mice fed a HFC diet. In addition, the efficacy of oat fiber is greater than wheat bran fiber in normalizing these metabolic disorders and pathological profiles.

Obesity is a key component of metabolic syndrome, a cluster of risk factors associated with increased risk of diabetes and cardiovascular morbidity^1^,^2^. In disease etiology, fat stored in non-adipose tissue, as so-called ectopic sites, is an important factor^3^. Elevated hepatic fat content and the release of free fatty acids (FFAs) from dysfunctional adipocytes results in lipotoxicity, caused by the accumulation of triglyceride-derived toxic metabolites in ectopic tissues (such as liver, muscle, pancreatic beta cells) and subsequent activation of inflammatory pathways, cellular dysfunction, and lipoapoptosis^4^,^5^. Recent investigation has shown that sterol-regulatory element binding proteins (SREBPs) are substrates of mitogen-activated protein kinases, and proposed that SREBP-1 might play an important role in the development of cellular features belonging to hepatic lipid accumulation and liver lipotoxicity^6^. FFAs and triglyceride (TG) syntheses in mammals is regulated by SREBP-1c, and the key regulator of SREBP activity is upregulated by activation of peroxisome proliferator-activated receptor alpha (PPARa). PPARa activators regulate obesity in rodents by increasing hepatic fatty acid oxidation and decreasing circulating TG levels responsible for adipose cell hypertrophy and hyperplasia^7^.

Metabolic syndrome and other obesity-associated diseases are managed by increased consumption of dietary fiber^8^,^9^,^10^. And dietary fiber is associated with decreasing appetite and body weight^11^,^12^. However, dietary fiber intake in modern societies is low, for example in China, the average intake was reported to be 12 g/d in adults according to national nutrition and health survey in 2002. Dietary fiber comprises a complex group of substances, it is of great interest to find dietary fiber with the most favorable effects should be identified and mechanisms of these effects should be elucidated. The type of dietary fiber corresponds to different physiological functions^13^. For instance, meta-analyses have shown that increased cereal dietary fiber reduces diabetes risk, by contrast, fruit and vegetable fiber is not significantly associated with diabetes risk^14^. As an exposure variable in nutritional research, cereal dietary fiber can be considered as a “black box,” because it contains molecular structures that may differ in the physiological potential^15^.Soluble fibers, mainly found in barley and oat, improve glycemia and insulin sensitivity of diabetic subjects^10^. Furthermore, oat β-glucan may lower plasma cholesterol levels^16^, by entrapping bile acids or reducing motility in the upper part of the intestinal tract^17^. Insoluble fibers, commonly found in wheat bran, increase transit time and fecal bulking^18^, the two parameters probably prevent colon cancer by diluting carcinogens and by reducing the time at which these carcinogens are in contact with epithelial cells.

Although adequate intake of cereal dietary fibers has proven metabolic and cardiovascular benefits, the molecular mechanisms remain still limited. Therefore, the purpose of the present study was to elucidate the roles of SREBPs and PPAR pathways in the pathogenesis of diet-induced liver lipotoxicity and dyslipidemia, and to explore most importantly the roles of cereal dietary fiber in the prevention of these metabolic disorders at molecular, cellular and intact animal levels. The efficacy of oat fiber and wheat bran fiber was also compared.

## Results

### Body weights, food and energy intake, and organ index

**Figure 1:**
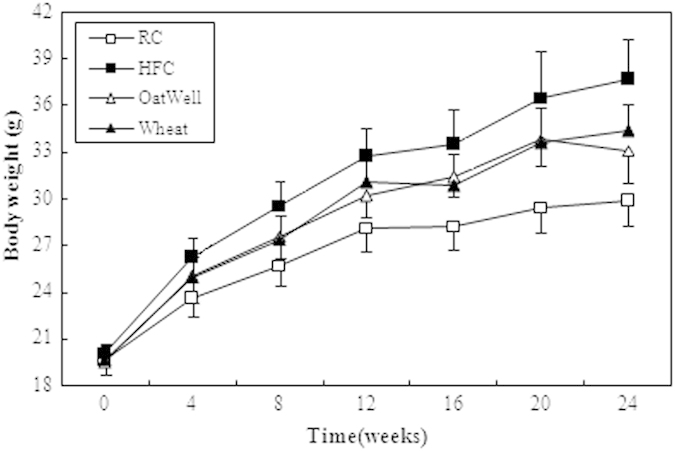
Effects of dietary fiber on body weights in C57BL/6J mice fed a high fat/cholesterol diet for 24 weeks. Each point is the mean ± SD of twelve mice. RC, reference chow diet group; HFC, high-fat/cholesterol diet group; OatWell, HFC plus 0.8% oat fiber group; Wheat, HFC plus 0.8% wheat bran fiber group.

At the baseline, no significant difference in body weights was observed among the four diet groups. On the third week, the body weights of HFC diet-fed mice (24.54 ± 1.08 g) were significantly greater than those RC diet-fed diet (22.70 ± 0.89 g) (p < 0.01). After 24 weeks of feeding, the body weights of HFC diet-fed mice were approximately 7.8 g heavier than RC diet-fed mice (Fig. 1), by contrast, OatWell or Wheat diet-fed mice exhibited considerably lower weight gain than HFC diet-fed mice (Table 1, Fig. 1). Although no significant difference in body weights was observed in both OatWell and Wheat diet groups, the average food intake and average energy intake in Wheat diet group were significantly higher than those in OatWell diet group (p < 0.05). Throughout the experimental period, animals in Wheat diet group showed a considerably higher food intake than animals in HFC and OatWell diet groups (p < 0.05). Animals in the two groups of HFC and OatWell showed a similar food intake pattern (p > 0.05). Animals in RC, HFC and OatWell diet groups did not significantly differ in energy intake (p > 0.05), by contrast, the Wheat diet group exhibited a considerably higher energy intake than the three other diet groups (p < 0.05) during the whole experimental period. The four diet groups showed similar organ indexes of the liver (p > 0.05). OatWell and Wheat diet groups displayed a significant decrease in the index of adipose tissues, including peritoneal and epididymal fats , compared with the HFC diet group, (p < 0.05). However, there was no significant difference in the two diet groups of OatWell and Wheat (p > 0.05).

### Histological analysis of the liver and adipose tissues

**Figure 2:**
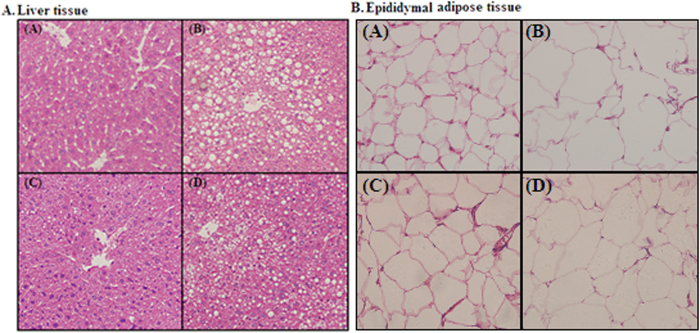
Histological examination of the liver tissue (100× ) and epididymal adipose tissue (400× ) of C57BL/6J mice following HE staining. (**A**) Reference chow diet group; (**B**) High fat/cholesterol diet group; (**C**) High fat/cholesterol diet plus 0.8% oat fiber group; (**D**) High fat/cholesterol diet plus 0.8% wheat bran fiber group.

HE-stained paraffin sections revealed normal hepatic architecture in clear hepatic lobule, radial liver cell cord, and clear hepatic sinusoid in the livers of the RC diet-fed mice. By contrast, pathological symptoms were observed in the livers of HFC diet-fed mice. With abundant and large lipid droplets in the cytoplasm, the liver cell appeared serious fatty degeneration and stromal inflammatory cell infiltration. After the mice were fed with cereal dietary fiber for 24 weeks, liver cell degeneration was lessened, and oat fiber was more effective in alleviating hepatic fatty degeneration than wheat bran fiber (Fig. 2A). As shown in Fig. 2B, the size and the volume of fat cells were considerably larger in the HFC diet group than in the RC diet group. By contrast, the size and volume of fat cells were reduced in cereal dietary fiber diet-fed mice, compared with those of the HFC diet-fed mice.

### Serum lipids profiles and HOMA-IR index

As shown in Table 2, HFC diet-fed mice exhibited an increase in TC (by 48.6%), TG (by 27.3%), FFAs (by 30.1%), glucose (by 47.7%) insulin (by 36.4%) concentration and HOMA-IR (by 82.9%) value, as compared with the RC diet-fed mice. However, oat fiber and wheat bran fiber attenuated HOMA-IR value and serum lipid profile, as indicated by reduced TC, TG and FFAs concentration, as compared with HFC diet group (p < 0.05). Serum TG, FFAs and glucose levels, and HOMA-IR value were considerably lower in the OatWell diet group than those in the Wheat diet group, but the difference between the two groups was not significant (p > 0.05). OatWell diet-fed mice showed significantly lower TC concentration thanWheat diet-fed mice (p < 0.05).

### Liver homogenate TG and FFAs

**Figure 3:**
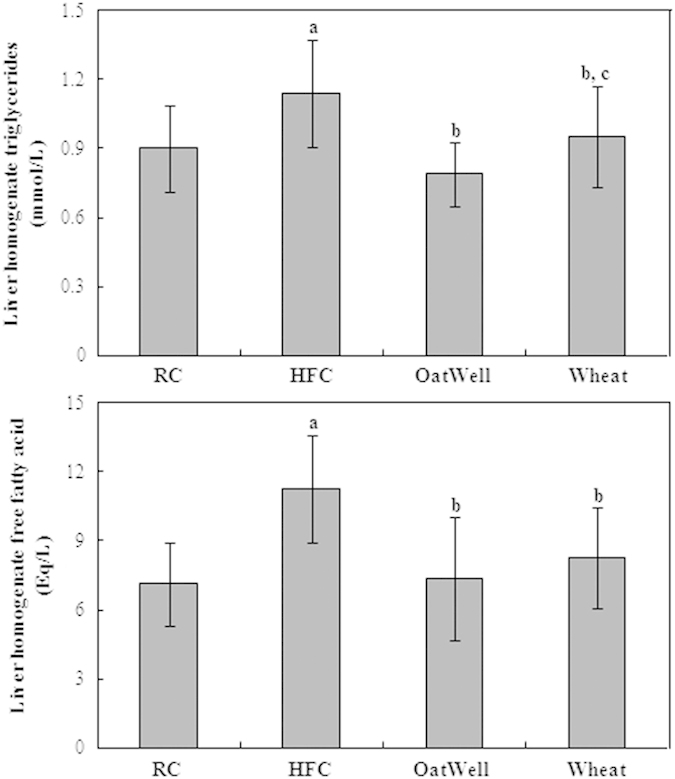
Effects of dietary fiber on liver homogenate TG and FFAs in C57BL/6J mice fed a high fat/cholesterol diet for 24 weeks. Bars represent the mean ± SD of twelve mice. RC, reference chow diet group; HFC, high-fat/cholesterol diet group; OatWell, HFC plus 0.8% oat fiber group; Wheat, HFC plus 0.8% wheat bran fiber group. ^a^p < 0.05, vs mice fed with RC diet. ^b^p < 0.05, vs mice fed with HFC diet. ^c^p < 0.05, vs mice fed with OatWell diet.

As shown in Fig. 3, liver homogenate TG and FFAs appeared similar changes to serum indexes. Liver homogenate TG and FFAs concentrations were significantly inhibited in OatWell and Wheat diet-fed mice after 24 weeks of feeding, compared with those in HFC diet-fed mice. Furthermore, a significantly lower liver homogenate TG was observed in the OatWell diet group than that in the Wheat diet group (p < 0.05).

### Serum adipocytokine levels

As shown in Table 3, the levels of serum adipocytokines, including IL-6, leptin and TNF-α were much higher in the HFC diet group than those in the RC diet group (p < 0.05). After 24 weeks of feeding, serum IL-6 and leptin concentrations decreased in the two groups of OatWell and Wheat compared with those in HFC diet-fed mice (p < 0.05). Serum TNF-α level was considerably lower in the Wheat diet group than that in the HFC diet group. However, serum TNF-α level did not significantly differ between the OatWell diet group and HFC diet group (p > 0.05).

### Protein expressions of SREBP-1, FAS and ACC

**Figure 4:**
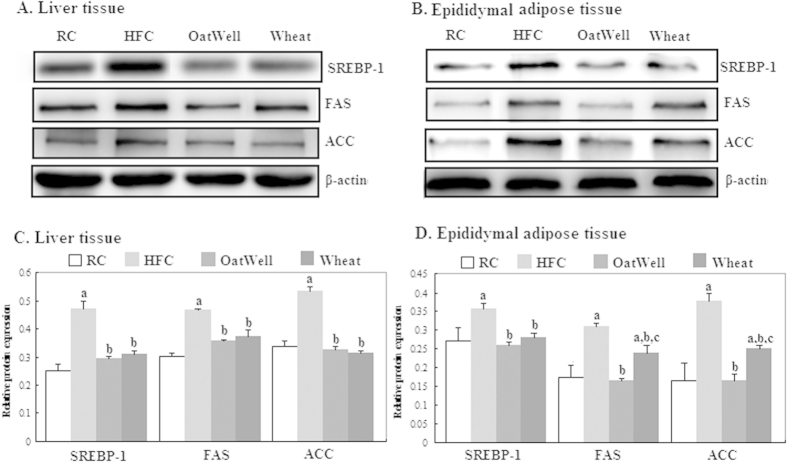
Effects of dietary fiber on protein expressions of SREBP-1, FAS and ACC in C57BL/6J mice fed a high fat/cholesterol diet for 24 weeks. The intensity of the bands was quantified by densitometric analysis and normalized with corresponding β-actin. Results are calculated as the mean ± SD of six mice. RC, reference chow diet group; HFC, high-fat/cholesterol diet group; OatWell, HFC plus 0.8% oat fiber group; Wheat, HFC plus 0.8% wheat bran fiber group; SREBP, sterol-regulatory element binding protein; FAS, fatty acid synthase; ACC, acetyl-CoA carboxylase. ^a^p < 0.05, vs mice fed with RC diet. ^b^p < 0.05, vs mice fed with HFC diet. ^c^p < 0.05, vs mice fed with OatWell diet.

Figure 4 shows the effects of oat fiber and wheat bran fiber on the protein expressions of SREBP-1, FAS, and ACC in the liver and epididymal adipose tissues of C57BL/6J mice, as determined by Western blot. On the basis of band densities (Fig. 4A,B), we found that the protein expressions of SREBP-1, FAS, and ACC in the liver and epididymal adipose tissues were significantly higher in the HFC diet group than those in the RC diet group (p < 0.05) (Fig. 4C,D). However, oat fiber and wheat bran fiber significantly minimized the increment in protein expression of SREBP-1, FAS and ACC after 24 weeks of feeding. The reduction in FAS and ACC expressions in the OatWell diet group was more notable than that in the Wheat diet group (p < 0.05).

### Protein expressions of PPARα and PPARγ

**Figure 5:**
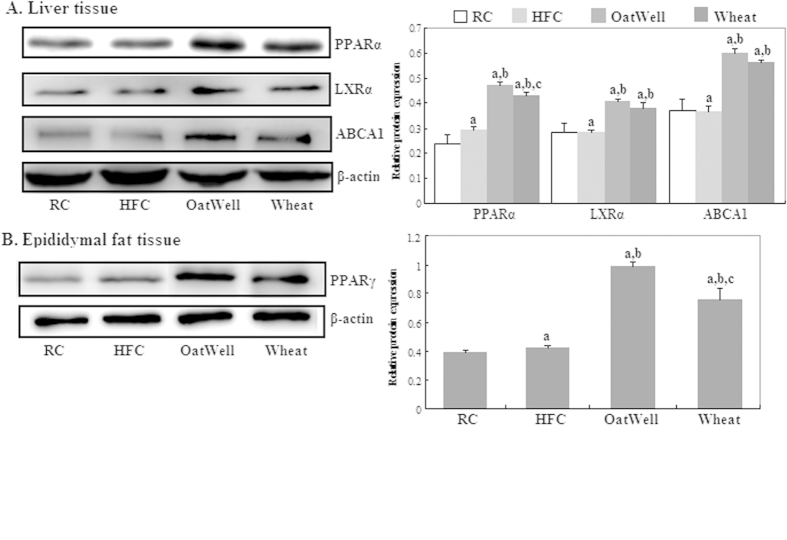
Effects of dietary fiber on protein expressions of PPARα, LXRα, ABCA1 and PPARγ in C57BL/6J mice fed a high fat/cholesterol diet for 24 weeks. The intensity of the bands was quantified by densitometric analysis and normalized with corresponding β-actin. Results are calculated as the mean ± SD of six mice. RC, reference chow diet group; HFC, high-fat/cholesterol diet group; OatWell, HFC plus 0.8% oat fiber group; Wheat, HFC plus 0.8% wheat bran fiber group; PPAR, peroxisome proliferator activated receptor; LXRa, liver X receptor alpha; ABCA1, ATP-binding cassette transporter A1. ^a^p < 0.05, vs mice fed with RC diet. ^b^p < 0.05, vs mice fed with HFC diet. ^c^p < 0.05, vs mice fed with OatWell diet.

We evaluated the protein expressions of PPARα, LXRα and ABCA1 in the liver tissue (Fig. 5A) and PPARγ (Fig. 5B) in the epididymal adipose tissue by Western blot. The protein expression levels of PPARα, LXRα, ABCA1 and PPARγ considerably increased after cereal dietary fiber was administered to HFC diet-fed C57BL/6J mice for 24 weeks (p < 0.05). No differences were detected in protein expressions of LXRα and ABCA1 between the two groups of OatWell and Wheat. Furthermore, the protein expressions of PPARα and PPARγ were much higher in the OatWell diet group than that in the Wheat diet group (p < 0.05).

## Discussion

Dietary fiber is deemed to be a key component in healthy eating^19^, which is recommended, alone or in combination with other lipid-lowering regimens. In the present study of experiment mode, our major new findings are two-fold: first, cereal dietary fibers in general normalized the expressions of SREBP1 and PPAR as well as other accompanied disorders (e.g. obesity, inflammatory biomarkers) in HFC diet-induced liver lipotoxicity and dyslipidemia in C57BL/6J mice; second, when these effects were compared, oat fiber could alleviate liver lipotoxicity and lipid profiles more effectively than wheat bran fiber.

Similar to previous finding, our results revealed that HFC diet increased body weights, serum TC and HOMA-IR value, and serum and liver homogenate TG and FFAs levels, consequently causing the occurrence of insulin resistance and liver lipotoxicity^20^,^21^,^22^. HFC diet also increased the concentrations of adipocytokines including IL-6, leptin and TNF-α. With cereal dietary fiber (oat fiber or wheat bran fiber) supplemented to HFC diet, cereal dietary fiber elicited beneficial effects against HFC diet-induced liver lipotoxicity by inhibiting TG accumulation in the liver and by decreasing serum and liver homogenate FFAs concentrations, as well as IL-6, leptin and TNF-α concentrations. Although no significant difference in body weights was observed between the two diet groups of OatWell and Wheat, the average food intake and the average energy intake in the Wheat diet group were significantly higher than those in the OatWell diet group. We concluded that wheat bran fiber was more effective in reducing body weights than oat fiber. The main reasons of high food intake in the Wheat diet group included the following points: first, we did not control the food intake during the whole experimental periods; second, animals of Wheat diet group had a big physical activity relative to the three other diet groups. Obesity was resulted from a prolonged energy imbalance. Enengy expenditure was a very important index. It was with regret that we did not measure enengy expenditure because of our limited experimental conditions.

Fiber-rich foods, particularly those with dietary fiber from cereals and whole grains, can help reduce subjective appetite and energy intake. Cereal dietary fiber may also help maintain or reduce body weight by promoting satiation and lowering caloric intake^23^, decreasing macronutrients absorption^24^, slowing the rate of starch digestion, and stimulating the release of gut hormones^25^. Important epidemiological evidence has further demonstrated that cereal dietary fiber is associated with decreased body weight or weight gain^26^. In addition, oat fiber rich in soluble fiber was more effective in decreasing serum TC, compared with wheat bran fiber in C57BL/6J mice fed with a HFC diet. This decrease may be accounted for a mechanism which soluble fiber is fermented by intestinal microflora, and fermentation could then modify the short chain fatty acids production, thereby reducing the acetate and increasing the propionate synthesis. This process consequently reduces endogenous synthesis of cholesterol and FFAs^27^. In a meta-analysis, the effect of soluble fiber on TC concentration was more effective than insoluble fiber^28^. The latest recommendations from the panel of American experts on cholesterol control (National Cholesterol Education Program III, 2002) highlighted the benefits of added variable quantity of soluble fiber and phytosterols to the diet as a primary or secondary prevention strategy to delay pharmacological treatment or to avoid unnecessary increases in the doses of hypolipidemic agents. Consequently, intake of foods high in cereal dietary fiber provides benefits regarding body weight, lipid profiles and cardiovascular risk^29^.

Lipotoxicity is a result of an imbalance between lipid uptake and utilization. The abnormal metabolism of FFAs and their derivatives is the main cause of intracellular lipotoxic injury^5^. Fat accumulation in the liver is generally associated with the cluster of metabolic abnormalities related to metabolic syndrome^30^. Furthermore, increased lipid droplet content is strongly correlated with insulin resistance, an early hallmark of developing type 2 diabetes mellitus^31^,^32^. Increasing circulating FFAs and causing lipid overloading induce a lipotoxic state in non-adipose tissues that gives rise to insulin resistance^33^. Our results exhibited that hyperlipidemic mice fed with cereal dietary fiber, maintain almost normal FFAs concentrations and improve insulin resistance. This result indicated minimal hepatic steatosis. Oat fiber was more effective in lowering the levels of serum and liver homogenate FFAs, and increasing insulin sensitivity than wheat bran fiber. Dietary fiber elicits hormonal effects by reducing insulin secretion. Dietary fiber also causes metabolic effects by increasing fat oxidation and decreasing fat storage because of increased satiety^34^.

SREBP-1 is a critical gene transcription factor that plays an important role in regulation of fatty acid synthesis and lipid metabolism, particularly in liver tissue^35^,^36^. Activated SREBP-1 inhibits lipogenesis by regulating the lipogenic enzymes of FAS and ACC^37^,^38^. In our study, HFC diet increased SREBP-1 expression, by contrast, cereal dietary fiber supplementation reversed this effect. Meanwhile, protein expressions of FAS and ACC were reduced in the two diet groups of OatWell and Wheat. In the epididymal adipose tissue, oat fiber was more effective in reducing FAS and ACC protein expressions than wheat bran fiber. The SREBP-1c gene promoter contains response elements for insulin, glucagon and LXR^36^. Furthermore, the SREBP-1c gene is potently activated by LXRα, which forms heterodimers with retinoid X receptors (RXR)^39^. LXR/RXR can also bind the SREBP-1c promoter and induce the SREBP-1 activation of FAS^40^. Our results showed that cereal dietary fiber downregulated the protein expression of LXRα, compared with HFC diet.

In addition, PPARs are members of the nuclear receptor superfamily that may be involved in the pathogenesis of liver lipotoxicity. PPARs are also key regulators of adipogenesis^41^,^42^,^43^. Our results suggested that PPARγ agonists are among the most potential treatments that decrease hepatic lipid accumulation and attenuate inflammatory responses^41^. The key regulator of SREBP activity is upregulated by activation of both PPARa and PPARγ. This study also showed that the protein expressions of PPARa and PPARγ were much higher in the HFC diet group than in the RC diet group. Inoue *et.al.* also demonstrated that a high-fat diet can increase PPARa and PPARγ expressions^44^. Compared with a HFC diet, cereal dietary fiber significantly increased the relative expressions of PPARa and PPARγ in the liver or in the epididymal adipose tissues. The effect was increased to a greater extent in the OatWell diet group than in the Wheat diet group. PPARа can reduce liver lipid accumulation and alleviate liver lipotoxicity^43^, and PPARγ can enhance insulin action and increase FFA oxidation^45^. The activation of the nuclear transcription factors PPARа and PPARγ can regulate the transcription of genes, such as SREBP-1, LXRа, ABCA1, involved in fatty acid synthesis and insulin sensitivity, in order to accelerate the β-oxidation of fatty acids and the clearance of lipids. This process can also alleviate liver lipotoxicity by decreasing TG and FFA contents in the liver tissue. These findings provided significant insights into the potential of cereal dietary fiber on suppression of lipid profiles and liver lipotoxicity induced by a HFC diet in C57BL/6J mice.

In conclusion, the present study provides further evidence indicating that cereal dietary fiber can prevent obesity-related liver lipotoxicity and ameliorate lipid profiles in C57BL/6J mice fed with a HFC diet, by increasing PPARα and PPARγ protein expression, and by decreasing SREBP-1 protein expression. It is more effective on reducing lipid profiles and improving liver lipotoxicity in the OatWell diet group than that in the Wheat diet group. Nevertheless, the precise molecular mechanisms of cereal dietaty fiber against obesity-related liver lipotoxicity and dyslipidemia in-depth remain to be elucidated, in order to promote the proofs of cereal dietary fiber in preventing metabolic syndrome and other obesity-associated diseases.

## Methods

### Animals and ethics statement

All of the animal studies were pre-approved by the Soochow University Animal Welfare Committee (Suzhou, China) and by China Zoological Society. All procedures of the investigation were in accordance with the principles outlined in China Practice for the Care and Use of Laboratory Animal. Seven-week-old male C57BL/6J mice were obtained from SLAC Laboratory Animal Company (Shanghai, China). They were housed in standard cages (3–5 mice per cage) at a constant temperature of 22 ± 2 °C with 55 ± 5% relative humidity and an artificial 12-h light-darkness cycle. Given that each group contained 12 mice, mice were housed in three cages, and allowed access to water and food ad libitum during the whole experimental periods.

### Experimental diets and design

After two weeks acclimatization to the laboratory conditions, animals were weighted and randomly assigned to four diet groups (n = 12), and maintained on respectively experimental diets for 24 weeks. The animals were given a reference chow (RC) diet, or a high fat/choleserol (HFC) diet, or HFC plus 0.8% oat fiber containing 22% β-glucan (OatWell) (DSM Nutritional Products Ltd, Shanghai, China) diet, or HFC plus 0.8% wheat bran fiber (Wheat) (Aote Food Science and Technology Company, Shanxi, China) diet. The RC diet contained 3.90 kcal/g with 20.8%, 67.7% and 11.5% calories from protein, carbohydrates, and fat. The HFC diets, which was used as a reference diet in the present study and known to induce obesity and dyslipidemia, were obtained from Research Diets, Inc (D12451 + 1% cholesterol) and contained 4.77 kcal/g with 19.6%, 34.4% and 46% calories from protein, carbohydrates, and fat. The OatWell and Wheat diets respectively contained 4.74 kcal/g and 4.75 kcal/g. The percentage and type of fat from the two diets were almost consistent with HFC diet. Body weights and average food intakes were routinely recorded weekly. The average calories intake were calculated by food intake and calories content in the four different diet groups.

### Blood and tissue collection

Blood samples were taked from the angular vein after 12-h of fasting, at the start of the feeding period. At the age of 24 weeks, overnight fasted animals given mild ether anesthesia, were killed after collecting the blood sample. Serum was separated by centrifugation at 3000rpm for 10 min and stored at −80 °C freezer until analyzed. The liver and epididymal and perirenal adipose tissues were immediately collected, weighted, frozen in liquid nitrogen and then stored at −80 °C freezer for further analysis.

### Histological examination of liver and adipose tissues

Histological examination of the liver and epididymal adipose tissue samples were carried out with HE staining method described by Osman *etal.*^46^. The samples were fixed in 10% neutral buffered formalin for 6-h and dehydrated as standard before embedding in paraffin wax sections (4 μm) were cut and mounted on polylysine-handled glass slides and HE staining was performed.

### Biochemical analysis

Serum levels of total cholesterol (TC), TG and glucose were determined by enzymatic methods using commercial kits (Applygen Technologies Inc, Beijing, China). Serum FFAs were determined by ACS-ACOD method according to the specification strictly (Wako Pure Chemical Industries, Ltd, Osaka, Japan). Serum insulin was measured by a Elisa kit (Mercodia, Sweden) and homeostasis model assessment of insulin resistance (HOMA-IR) was calculated according to the reported formula^47^. Adipocytokines from fasting serum including interleukin-6(IL-6), leptin, and tumor necrosis factor-alpha (TNFα) were measured with Luminex^®^ xMAP Technology (Merck Millipore Bioscience, CA, USA).

### Hepatic lipid analysis

Lipid levels of TG in the liver were analyzed with commercialized kits (Applygen Technologies Inc, Beijing, China) and normalized to protein levels (Beyotime Institute of Biotechnology, Nantong, China). Liver homogenate FFAs were determined by ACS-ACOD method and normalized to protein levels.

### Western blot analysis

The liver or epididymal adipose tissue samples were homogenized in lysis buffer (1:10wt/vol). The protein concentration was determined according to the BCA Protein assay (Beyotime Institute of Biotechnology, Nantong, China). Equal amounts of protein (30–50 μg) were loaded to a 12% SDS-polyacrylamide gel and then subsequently transferred to PVDF membrane (EMD Millipore Corporation, CA, USA) by electrophoretic transfer. Subsequently, nonspecific binding sites were blocked with 5% fat-free dry milk in phosphate-buffered saline containing 0.1% Tween-20 for 1-h at room temperature and blots were incubated overnight at 4 °C with anti-SREBP-1 (1:500; Abcam), anti-LXRα (1:1000; Abcam), anti-ACC (1:1000; Cell Signaling Technology), anti-FAS (1:1000; Cell Signaling Technology), anti-PPARγ (1:1000; Cell Signaling Technology), anti-PPARα (1:2000; EMD Millipore) and anti-ABCA1 (1:1000; EMD Millipore) antibodies. After washing three times, the antigen-antibody complexes were visualized for 1-h at room temperature with Peroxidase AffiniPure goat anti-mouse or anti-rabbit IgG antibody (1:2000; Jackson ImmunoResarch Laboratories). Antibody reactivity was detected by chemiluminescene ECL Detection Systems (EMD Millipore). Blots were performed at least three times to confirm the reproducibility of the results. The intensity of the bands was normalized using each corresponding β-actin density as an internal control.

### Statistical analysis

All data are expressed as the mean ± standard deviation (SD). All the analysis were performed using SPSS version 17.0 statistical analysis package (SPSS Inc., Chicago, IL, USA). The significance of difference among the four dietary groups was assessed by analysis of one-way variance (ANOVA), followed by the Turkey post hoc test. A p-value < 0.05 was considered significant.

## Additional Information

**How to cite this article**: Han, S. *et al.* Dietary fiber prevents obesity-related liver lipotoxicity by modulating sterol-regulatory element binding protein pathway in C57BL/6J mice fed a high-fat/cholesterol diet. *Sci. Rep.* **5**, 15256; doi: 10.1038/srep15256 (2015).

## Figures and Tables

**Table 1:** Effects of dietary fiber on average food and energy intake, weight gain, organ index in C57BL/6J mice fed a high fat/cholesterol diet for 24 weeks.

	RC	HFC	OatWell	Wheat
Food intake (g/day)	3.14 ± 0.23	2.57 ± 0.22^a^	2.55 ± 0.30^a^	2.80 ± 0.34^a^^,^^b^
Energy (kcal/day)	12.26 ± 0.89	12.25 ± 1.08	12.09 ± 1.44	13.29 ± 1.63^a^^,^^b^^,^^c^
Weight gain (g)	10.21 ± 1.46	17.55 ± 2.60^a^	13.63 ± 2.01^a^^,^^b^	14.73 ± 1.84^a^^,^^b^
Relative liver weight (mg/g body weight)	39.06 ± 2.58	38.05 ± 1.65	38.14 ± 3.67	38.49 ± 3.67
Relative fat weight (mg/g body weight)	18.94 ± 7.63	60.23 ± 12.60^a^	39.22 ± 14.57^a^^,^^b^	48.20 ± 19.40^a^^,^^b^

Data are expressed as the mean ± SD (n = 12).

^a^p < 0.05, vs mice fed with RC diet.

^b^p < 0.05, vs mice fed with HFC diet.

^c^p < 0.05, vs mice fed with OatWell diet.

**Table 2:** Effects of dietary fiber on lipid profiles and HOMA-IR index in C57BL/6J mice fed a high fat/cholesterol diet for 24 weeks.

	RC	HFC	OatWell	Wheat
TC (mmol/L)	1.81 ± 0.20	2.69 ± 0.45^a^	1.82 ± 0.39^b^	2.21 ± 0.50^a^^,^^b^^,^^c^
TG (mmol/L)	1.39 ± 0.33	1.77 ± 1.63^a^	1.28 ± 0.29^b^	1.39 ± 0.41^b^
FFAs (Eq/L)	1.63 ± 0.24	2.12 ± 0.38^a^	1.78 ± 0.38^b^	1.83 ± 0.40^b^
Glucose (mmol/L)	5.56 ± 2.78	8.21 ± 2.06^a^	7.10 ± 1.47^b^	7.56 ± 1.11^a^^,^^b^
Insulin (μg/L)	0.55 ± 0.09	0.75 ± 0.21^a^	0.57 ± 0.13^b^	0.63 ± 0.15
HOMA-IR	3.51 ± 0.81	6.42 ± 2.00^a^	4.05 ± 1.31^b^	4.31 ± 0.87^b^

Data are expressed as the mean ± SD (n = 12).

^a^p < 0.05, vs mice fed with RC diet.

^b^p < 0.05, vs mice fed with HFC diet.

^c^p < 0.05, vs mice fed with OatWell diet.

**Table 3:** Effects of dietary fiber on serum adipocytokine levels of IL-6, leptin and TNF-α in C57BL/6J mice fed a high fat/cholesterol diet for 24 weeks.

	RC	HFC	OatWell	Wheat
IL-6 (pg/mL)	3.64 ± 1.80	7.93 ± 3.16a	5.72 ± 1.81^a^^,^^b^	5.14 ± 1.74^b^
Leptin (ng/mL)	0.73 ± 0.42	7.14 ± 3.44^a^	4.27 ± 1.37^b^	4.79 ± 2.26^b^
TNF-α (pg/mL)	2.67 ± 0.86	3.86 ± 1.45^a^	3.41 ± 0.83	2.89 ± 0.85^b^

Data are expressed as the mean ± SD (n = 12).

^a^p < 0.05, vs mice fed with RC diet.

^b^p < 0.05, vs mice fed with HFC diet.

^c^p < 0.05, vs mice fed with OatWell diet.
